# Impact of COVID-19 lockdowns on house sparrows: Comparative study from an Indian context

**DOI:** 10.1371/journal.pone.0289548

**Published:** 2023-08-15

**Authors:** Sudeep R. Bapat, Sreeranjini T. M.

**Affiliations:** 1 Department of Operations Management and Quantitative Techniques, Indian Institute of Management, Indore, India; 2 School of Mathematics and Statistics, University of Hyderabad, Hyderabad, India; Forest Research Institute Dehradun, INDIA

## Abstract

Due to the outbreak of COVID-19, the last couple of years have been drastic in terms of human behavioural patterns. The pandemic has taught us key lessons about crisis, communication and misinformation. People were forced to stay at home for a very long duration because of the strict lockdown measures imposed by governments all over the globe. India was no exception, wherein the Indian government imposed several very strict lockdowns all across the country, which restricted human activities and their social behaviours. However, such restrictions were seen to have a positive impact on environment and ecology. In this paper, we aim to study the changes in House sparrow sightings, as a result of the lockdowns. It is postulated that the lockdowns give rise to increased House sparrow numbers, which we try to argue, using appropriate exploratory analysis and statistical modelling. We apply a specific “zero-inflated Poisson” regression model in this regard.

## 1 Introduction

**Fig 1:**
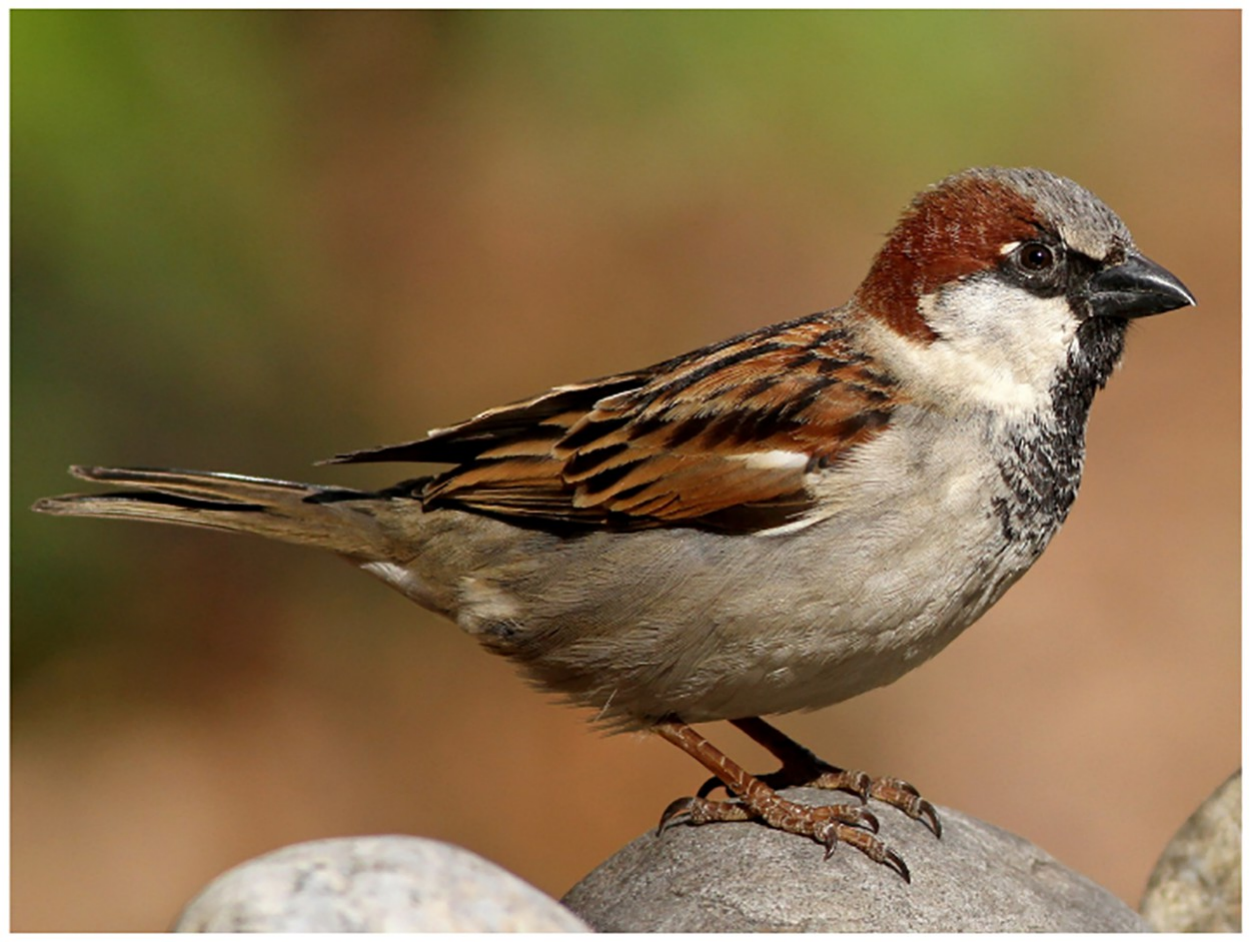
A typical house sparrow. (Image provided by eBird (www.ebird.org) and created on [16/04/2015]).

The “House sparrow”, scientifically known as *Passer domesticus* is one of the most commonly found birds across India. Since a very long time, the relationship between human beings and sparrows has been harmonious. They are easily observable in windows and balconies of houses, which shows how well they have adapted with the human lifestyle. A typical house sparrow sighted in India is as seen in Fig 1. But since the starting of the 21st century, their population has been threatened and the population curve is also dipping. Several reasons have been cited for this decline, one of them being the increasing urbanization and pollution. One may refer to [1], where the authors indeed claim a decline in their number, according to a study conducted by the Indian Council of Agricultural Research (ICAR). Even though the cause of decline was not known specifically, it is believed that it was due to the non-availability of nests, due to modernization and urbanization. Organizations like Nature Forever Society (NFS) have adopted several measures to conserve sparrows like “Common Bird Monitoring of India”, “World Sparrow Day”, “Project Save Our Sparrows”, among others. In literature, there have been many research articles published in recent times, which also suggest a decline in the number of house sparrows. Some of these include, an article by [2], or [3], where the authors conjectured that the house sparrow is on the verge of decline due to urbanization which leads to the unavailability of suitable nesting sites. [4] studied the impact of urbanization on house sparrow distribution, particularly in the city of Kolkata, India. A few other related articles which one may refer to are by [5], where the authors surveyed the house sparrow decline in West Bengal, [6], who analysed the effect of COVID-19 lockdowns on the diversity of bird and mammals in Nepal, or [7], where they studied the effect of urbanization on the bird diversity in Kerala, India.

In this paper, we focus on a related issue, that of studying the effects of COVID-19 lockdowns on house sparrow counts. As discussed earlier, the pandemic impacted the entire world drastically. Human behaviours and social interactions were hampered, mainly because of the strict lockdowns imposed by governments across the globe. However, such lockdowns were a boon to the ecology and the environment. The wildlife sightings increased after COVID hit and especially during lockdowns. One belief is that since the humans were not allowed to roam much, they could spend more time observing and recording such sightings. There have already been a lot of literature stating that the lockdowns indeed impacted the wildlife in a positive way. One may refer to [8], where the authors investigated how stay-at-home orders affected data submitted by birdwatchers in Italy, Spain and the United Kingdom. [9] found that counts of several focal bird species changed in pandemic-altered areas, usually increasing in comparison to prepandemic era. [10] assessed changes in reproductive success of great tits *(Parus major)* at two urban habitats, due to the COVID pandemic. [11] studied the birds’ response to the population lockdown by using bird records collected by a citizen science project in northeastern Spain.

Specific in the context of this paper, a brief overview of how things evolved in India is as follows: On January 30th, 2020, India’s first ever COVID case was reported in the state of Kerala. As the number of COVID cases increased, the Prime Minister of India announced a nationwide lockdown on the midnight of March 24th, which lasted initially for 21 days. Further, it was extended up to May 31st, 2020. The movement and activities of the citizens got restricted as a result of this imposition of lockdown. This sudden pause in the human activities also astonishingly brought down the air and noise pollution levels. This gave us a rare opportunity to carry out a comparative study about how the less crowded cities affected the lives of birds like house sparrows. One of the assumptions is that the strict “stay at home” orders might have brought more people into birdwatching and the time spent on such activities might have increased. Thus, we hypothesized that there could be an increase in the daily number of observations after the imposition of lockdown.

The structure of the paper is as follows: Section 2 provides the methodology used for the analysis, along with a brief description of the dataset. Section 3 covers the statistical analysis and modelling of the sparrow counts using appropriate count regression models, while Section 4 includes a brief discussion.

## 2 Methodology

For the purpose of our study, we downloaded data on house sparrow citing in India from eBird [12], which is one of the largest biodiversity science projects with more than 100 million bird sightings contributed annually by eBirders around the world. The data contained house sparrow checklists from 01/01/2018 to 30/11/2021 collected by bird observers across India. There were 187898 observations across 29 variables, including observation count, county, state, locality (hotspot or personal), date of sighting, time of sighting, duration, number of observers in the group etc. Initially, to analyse the change in the number of bird sightings during the lockdown as compared to the previous years, we performed a yearly comparison of the bird counts from 2018 to 2021. Since the data contained multiple sightings for the same day, for the ease of analysis, we first aggregated the different entries of the same day to form ‘daily bird count’ and then compared it year wise using various descriptive measures and graphical analysis. Basic boxplots and histograms revealed the presence of outliers in the data, which were removed, separately for each year, using the standard approach of judging the interquartile range (if an observation is 1.5 times the interquartile range more than the third quartile (Q3) or 1.5 times the interquartile range less than the first quartile (Q1), it is considered an outlier). Additionally, we also divided the timeline around COVID lockdown into five phases as follows:

- Phase 1: before first lockdown (before 24/03/2020)
- Phase 2: during first lockdown (24/03/2020 to 31/05/2020)
- Phase 3: before second lockdown (01/06/2020 to 04/04/2021)
- Phase 4: during second lockdown (05/04/2021 to 15/06/2021)
- Phase 5: after second lockdown (16/06/2021 to 31/11/2021)

We then conducted a phase-wise comparison of the daily bird count to understand if there is any variation in bird sightings in these time periods. We outlined the differences between bird counts by considering the phases in terms of several lockdowns which the government of India imposed, to curb the rising COVID cases. As discussed before, a belief was that due to the strict lockdowns, the number of sparrows increased, as a result of decreased human activities and all forms of pollution. We focused on the five phases, which are denoted as ‘before’, ‘lock1’, ‘after1’, ‘lock2’, and ‘after2’ for convenience. A point to note is that the two lockdowns namely, first and second, coincided with the first and second COVID waves, which also had a global effect. However, the sample sizes (number of days) under these different phases were not comparable, but we atleast spotted the differences visually.

Since the occurrence and/or detectability of birds could change throughout the hours of the day, we chose two specific times: around sunrise (4 am to 7 am) and around sunset (5 pm to 7 pm) and aggregated the number of daily observations in these two times. This provided us with an idea as to how the sparrow sightings are distributed during the two most sought after times for engaging in bird watching for any given day. To carry out this comparison, we adopted the following approaches. The first idea was to consider only the observations recorded around sunrise and sunset (separately) during the days of first lockdown in 2020 and the same dates in 2018. We purposefully picked 2018 as opposed to 2019 to ensure complete absence of any COVID traits. We also divided the timeline into two portions at the date 30/01/2020, named them as ‘before COVID’ and ‘after COVID’, and compared the daily bird count around sunrise and sunset.

Finally, we modelled the number of sightings during a particular time point by a Zero Inflated Poisson regression model, as we observed more zero sightings than expected in a regular Poisson model. We used the variables ‘locality type’ (hotspot or personal) and ‘starting hour of observation’ in the Poisson regression part, and the variable ‘Phase’ to model the excess zeroes. There have been similar attempts in the past to model the bird counts using a suitable regression technique. One may refer to [13], where the authors discuss about a hierarchical model applied on Christmas bird counts, or [14], where the authors have statistically evaluated bird populations in Akdoğan Lakes by means of Poisson and negative binomial regression models. We used a ZIP regression model using the same variables for predicting counts and phases of lockdown, for modelling the excess zeroes. A ‘Vuong’ test was used to compare the two models. The Vuong test can be employed to compare predicted probabilities of non-nested models and is generally used in comparing zero-inflated models with standard models. If the two models were not different, we would see a large p-value. ZIP regression is an example of the type of statistical model called as the ‘latent variable model’, where two or more processes occur together, which affect the response variable. In our case, there were two parts to this model. The first one was the standard Poisson regression part which is given by,

$$\text{log}\;\left(\lambda \right)=\beta_{0}+\beta_{1}\left(\text{locality}\;\text{type}\right)+\beta_{2}\left(\text{hour}\right),$$

while the other part modelled the excess zeroes and is given by,

$$logit\left(\alpha \right)=\beta_{0}+\beta_{1}\left(\text{locality}\;\text{type}\right)+\beta_{2}\left(\text{phase}\;\text{2}\right)+\beta_{3}\left(\text{phase}\;\text{3}\right)+\beta_{4}\left(\text{phase}\;\text{4}\right)+\beta_{5}\left(\text{phase}\;\text{5}\right),$$

where the five phases are as taken in Section 2. Note that we picked the base category to be ‘Phase 1’ (before first lockdown).

## 3 Results and discussion

**Fig 2:**
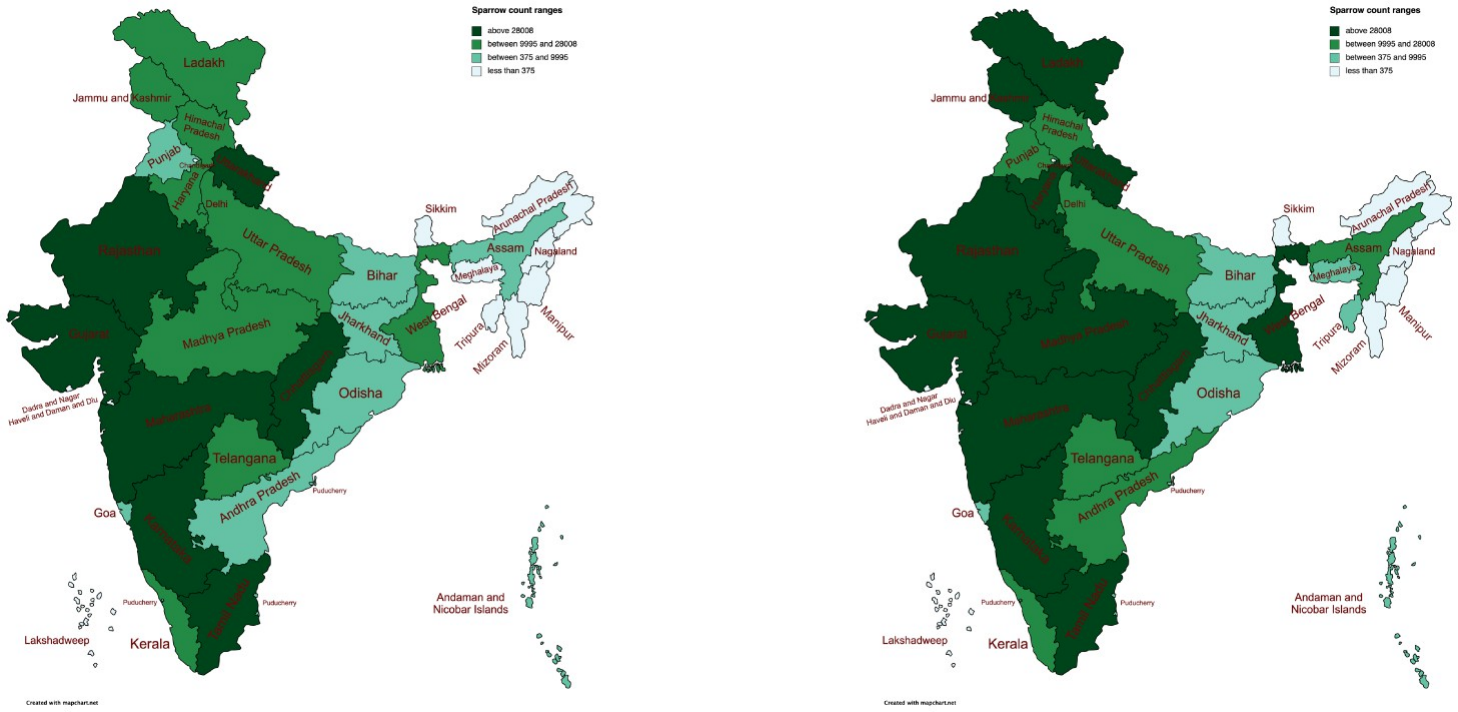
Side-by-side boxplots (top left), histograms (top right) and scatterplots (bottom) for the bird counts. Republished from mapchart.net (https://www.mapchart.net/india.html) under a CC BY license, with permission from Minas from Mapchart.

This section provides a wide array of exploratory and statistical techniques applied on different sets of bird counts, as a comparison and modelling approach. Before looking at some detailed analysis, we provide a glimpse of the spatial distribution of sparrows in India, according to the different states. Fig 2 contains side-by-side gradient maps of India showing the sparrow counts before COVID (before 30/01/2020) and after. One can note the clear increase in sparrow counts in the same states after the outbreak of COVID.

### 3.1 Year wise comparison

**Fig 3:**
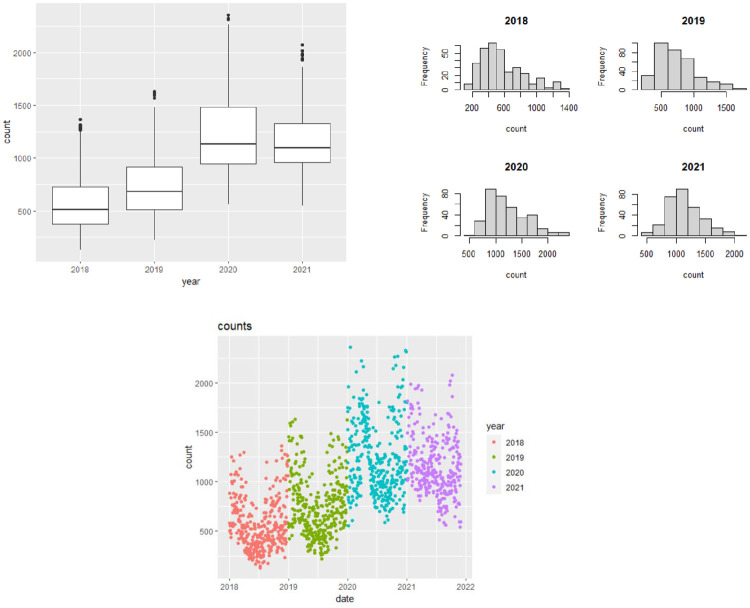
Side-by-side boxplots (top left), histograms (top right) and scatterplots (bottom) for the bird counts.

The first aspect was to conduct an appropriate year wise comparison of bird counts. To begin with, we assessed the trends in daily observation counts over time. We hence aggregated the daily observation count for each date from 1st Jan 2018 to 30th Nov 2021, into a single number. Fig 3 contains side-by-side boxplots, histograms and separate scatterplots showing the distribution of bird counts for each year from 2018 to 2021. Right away, one can notice the increase in the count in the years 2020 and 2021, as compared to the previous ones. Especially the histograms show that the counts become more symmetric for years 2020 and 2021. This gives a visual affirmation of the fact that sparrows were sighted more during COVID as compared to before.

Basic summary variables of the bird counts for each year is also presented in Table 1 for completeness. One can clearly note that the average bird count has indeed increased after the outbreak of COVID.

**Table 1:** Summary variables of bird counts for each year.

Year	No. of days	Min	*Q*_1_	Median	Mean	*Q*_3_	Max
2018	336	131	372	508	566.5	723.5	1365
2019	344	222	508	684	740.2	913.2	1629
2020	349	558	944	1130	1224	1481	2356
2021	305	544	956	1092	1149	1325	2075

Further, we also conducted a few other comparisons between the bird counts, by focusing on different time periods. The first comparison was between ‘before COVID’ and ‘after COVID’ sparrow counts. In order to do so, we considered the data from 01/03/2019 to 28/02/2020 as ‘before COVID’ and paired it with the same dates next year namely, 01/03/2020 to 28/02/2021, which was termed as ‘after COVID’. We conducted this comparison through a non-parametric approach, namely the Wilcoxon signed rank test. This is an ideal way to compare the locations of two matched samples. The hypotheses to test here are whether the true location shift equals zero or is less than zero. The corresponding p-value which was obtained was < 2.2 × 10^−16^, and we were able to conclude that the counts ‘after COVID’ were indeed substantially more.

**Fig 4:**
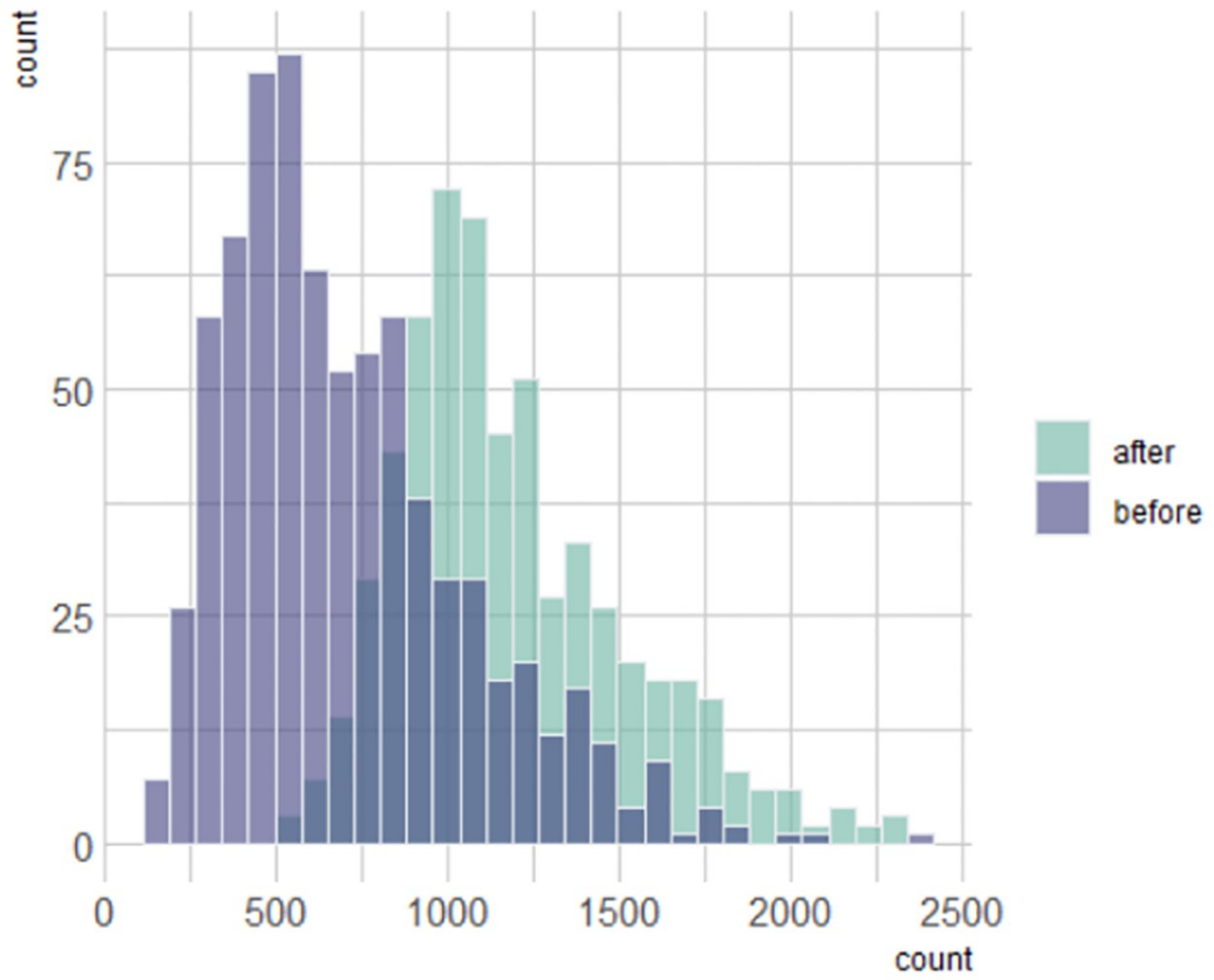
Comparative histograms for the bird counts before 30/1/2020 and after.

Another possible comparison was by looking at the entire available data before 30/1/2020, when the first COVID case was discovered in India, and that after 30/1/2020. Since both the samples can be considered to be independent, we applied another non-parametric approach namely, the Wilcoxon rank sum test. This test is capable of finding whether the locations for both the independent samples are equal or not. The obtained p-value was again <2.2 × 10^−16^, which concluded that the number of sparrow sightings increased after the very initial COVID outbreak in India. A more visual representation of this conclusion can be seen from the comparative histograms given in Fig 4. The y-axis gives the frequencies of the corresponding counts. One can easily note that for the ‘after’ group, higher frequencies correspond to larger bird counts.

### 3.2 Phase wise comparison

**Fig 5:**
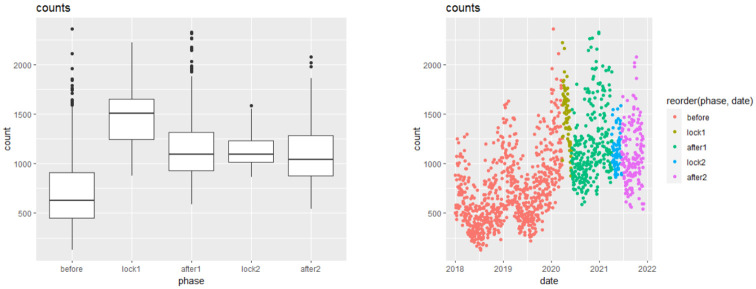
Side-by-side boxplots (left) and scatterplots (right) for the different phases.

Table 2 contains some basic summary variables for the bird counts in different phases. One can note that during both the lockdowns, even though the sample size is much less than other phases, the bird counts are still comparable to other time periods. This indicates the human tendency of engaging themselves in other interests, such as bird watching, during strict lockdowns, when they were not allowed to even step out of their houses. For a better understanding, Fig 5 contains side-by-side boxplots and scatterplots of the sparrow counts for the different phases. These present similar conclusions as were seen from the summary table. An interesting observation from the scatterplots is that during summers of every year, the bird counts were seen to be the least. This may correspond to the fact that people preferred staying at homes during peak summers and hence amounting to reduced sparrow sightings.

**Table 2:** Summary variables of bird counts for each phase.

Phase	No. of days	Min	*Q*_1_	Median	Mean	*Q*_3_	Max
before	754	131	449.8	625.5	710.9	906.5	2356
lock1	68	879	1246	1506	1469	1654	2220
after1	277	589	931	1092	1180	1316	2323
lock2	71	865	1014	1090	1142	1234	1584
after2	164	544	879.2	1043	1088.7	1285	2075

**Fig 6:**
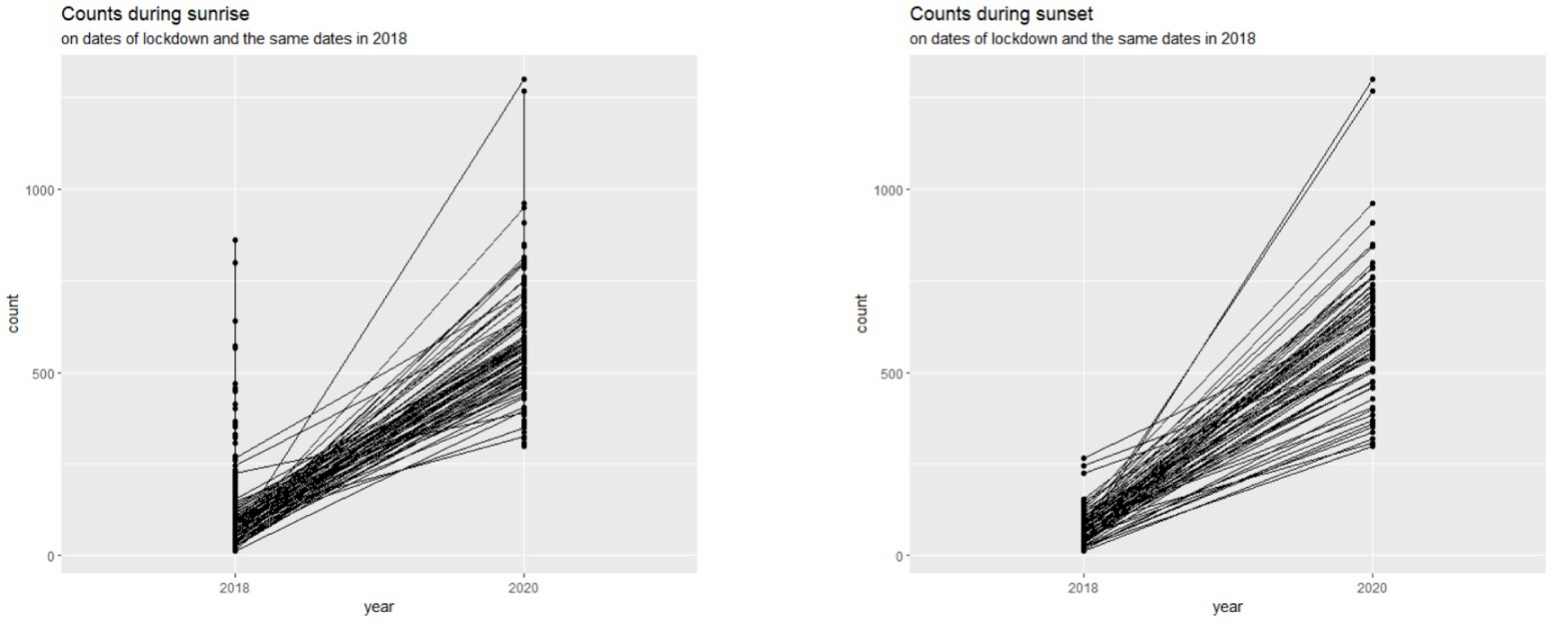
Comparitive scatterplots of bird counts during sunrise (left) and sunset(right).

Further, we observed that the mean daily bird counts at sunrise increased from 163.8 in 2018 to 597.8 in 2020 and that at sunset increased largely from 79.5 in 2018 to 620.2 in 2020. Fig 6 contains comparative scatterplots separately for sunrise and sunset, which show a connection between the sparrow counts for the same dates in 2018 and 2020 (during the first lockdown). One can note that during the first lockdown in 2020, more mass was towards the top of the vertical line, which indicates more number of higher bird count frequencies. The second idea was to understand the overall effects of COVID and lockdown. We thus adopted a similar technique which is seen before, to split the timeline at the date 30/01/2020, which amounts to ‘before COVID’ and ‘after COVID’ and compared the counts at sunrise and sunset. Table 3 contains some basic summary variables for this comparative study. One can clearly note that after the onset of COVID, the sparrow counts increased, both during sunrise and sunset.

**Table 3:** Summary variables of bird counts during sunrise and sunset.

Phase	Min	*Q*_1_	Median	Mean	*Q*_3_	Max
**Sunrise**						
before	8	100.2	167	220.3	277.2	1386
after	29	251	361	403.3	519	1341
**Sunset**						
before	8	77	116	150.8	177.8	1386
after	29	199	267.5	314.2	367.5	1326

### 3.3 Statistical modelling

**Fig 7:**
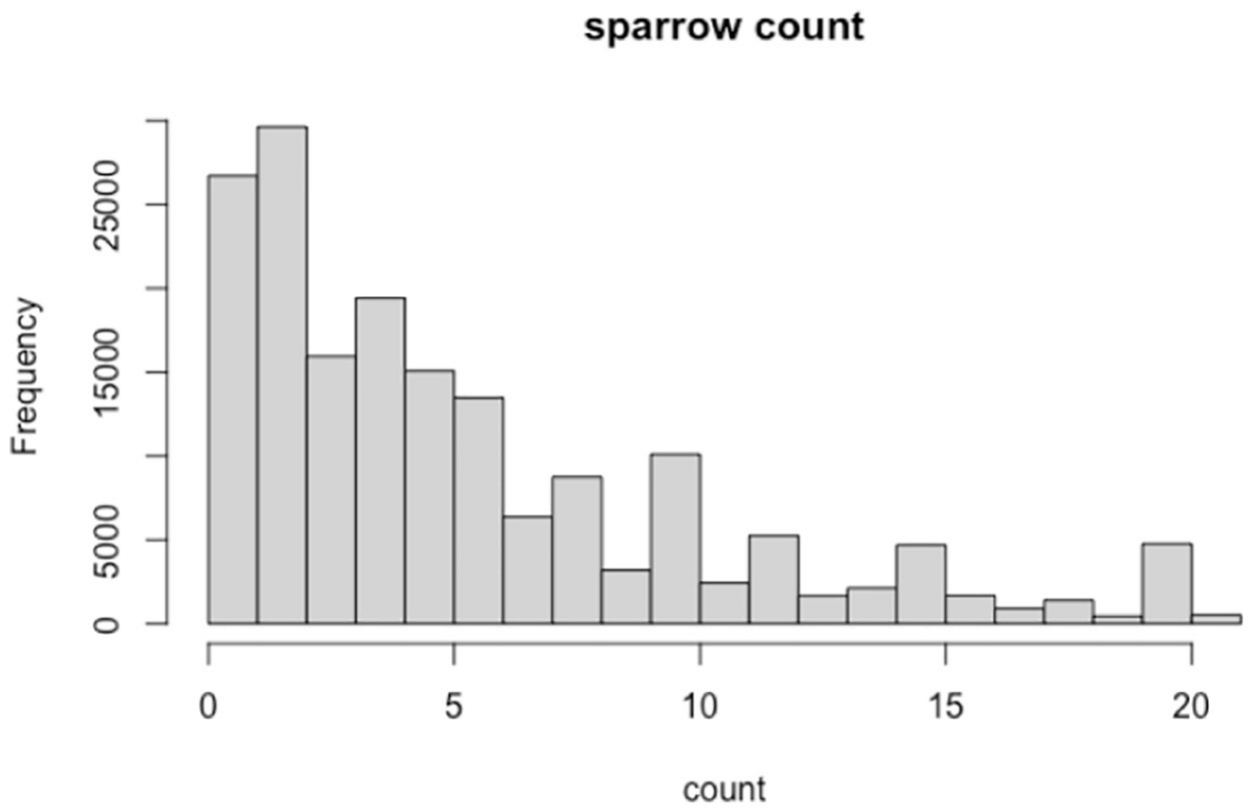
A simple histogram of bird counts indicating excess zeroes.

Since the sparrow sightings is a count data, we expected that a Poisson model would fit the observations better. We hence implemented suitable Poisson regression models, by taking a few related predictors and hence analysing their impact on the bird count. When we focused on the raw data (without daily aggregates), it contained more zeroes than expected. The excess zeroes could be generated by some other process than the Poisson process, like naturally low sparrow count of the location, lack of observer expertise, etc. Fig 7 contains a simple histogram for the bird counts coming from the raw data. One can clearly note the significant spike at 0. In this scenario, a Zero Inflated Poisson regression (ZIP regression) performed better, accounting for the excess zeroes. Firstly, we fitted a standard Poisson regression model to predict the count using two variables namely, “locality type” and “starting hour” of observation. Here the variable “locality type” refers to the code which defines the type of location used. The participants on eBird can plot specific locations on a map (P or Personal) or choose existing locations from a map (H or Hotspot).

In our case, the p-value for the Vuong test was small (< 2.2 × 10^−16^) which indicated that the ZIP model performed better than the standard Poisson model. Further, all the results of the ZIP regression model were in comparison to this base category. All the coefficients of the model in both count and inflation parts were statistically significant. For interpretation, we exponentiated all the coefficients of the model. It is observed that for a given starting hour of observation, a personal locality is expected to sight 1.143 times more birds than a hotspot. Also, for a given locality type, the bird count increased by a factor of 1.0012 with every 1 hour increase in the starting hour of observation. The variable ‘phase’ was used to predict excess zeroes in the model. The log odds of observing a true zero in phase 2 was lesser by -0.54770 compared to phase 1. It was lesser for phases 3, 4 and 5 by -0.42907, -0.51192 and -0.44459 respectively, compared to phase 1. This means that the probability of observing true zero in phase 1, i.e., before lockdown was 0.0642 while it was 0.0381, 0.0428, 0.0395 and 0.0421 for the next 4 phases respectively.

## 4 Conclusions

From this analysis one can note that the house sparrow sightings per day increased significantly after the imposition of lockdown. The increase in the daily number of bird counts could be mainly because more people might have started birdwatching during the lockdown as a new hobby or interest. Birdwatching might have helped them ease the hardships of COVID and the lockdown. This is supported by the fact that the count is 1.143 times more in a personal locality than in a hotspot, and thus, many people who reported might have done backyard birdwatching from their homes.

Another conclusion is that the sparrows were more visible due to quieter cities and towns with fewer human interactions. Before the onset of COVID, there are reports mentioning the declining sparrow population in India, and several individuals and organizations have taken several efforts to bring awareness about the protection of sparrows. Through the analysis in the present paper, we can sense that the sparrows might have been reviving. The sparrow counts at sunrise and sunset, which are the popular hours of the day for birdwatching, during lockdowns in 2020 and the same dates in 2018, saw a significant increase.

The probability of observing true zeroes decreased slightly in all the phases since lockdown. This also tells us that since lockdown, the number of people engaging themselves in bird watching and the duration of observations have increased. More sparrows were spotted in personal localities, indicating that since there were restrictions on movement, people watched birds more from their homes rather than travelling to nearby hotspots. We used the categorical variable ‘phase’ to predict excess zeroes since the excess zeroes might be produced by people observing for a shorter duration, and lockdown phases might affect the duration of the observations. The lockdown has also provided birdwatchers ample time to observe. The duration of observations has increased, and as a result, zero counts are observed lesser.
